# Biocontrol yeasts: mechanisms and applications

**DOI:** 10.1007/s11274-019-2728-4

**Published:** 2019-10-01

**Authors:** Florian M. Freimoser, Maria Paula Rueda-Mejia, Bruno Tilocca, Quirico Migheli

**Affiliations:** 1 Agroscope, Research Division Plant Protection, Müller-Thurgau-Strasse 29, 8820 Wädenswil, Switzerland; 2 Dipartimento di Agraria, Università degli Studi di Sassari, Viale Italia 39, 07100 Sassari, Italy; 3 Istituto Nazionale di Biostrutture e Biosistemi and NRD - Nucleo di Ricerca sulla Desertificazione, Università degli Studi di Sassari, Viale Italia 39, 07100 Sassari, Italy; 4 Department of Health Sciences, University “Magna Græcia” of Catanzaro, Viale Europa, 88100 Catanzaro, Italy

**Keywords:** Biological control, Microbial antagonism, Plant protection, Plant pathogens, Competition, Enzyme secretion, Toxin production, Volatiles, Mycoparasitism, Resistance induction

## Abstract

Yeasts occur in all environments and have been described as potent antagonists of various plant pathogens. Due to their antagonistic ability, undemanding cultivation requirements, and limited biosafety concerns, many of these unicellular fungi have been considered for biocontrol applications. Here, we review the fundamental research on the mechanisms (e.g., competition, enzyme secretion, toxin production, volatiles, mycoparasitism, induction of resistance) by which biocontrol yeasts exert their activity as plant protection agents. In a second part, we focus on five yeast species (*Candida oleophila, Aureobasidium pullulans, Metschnikowia fructicola, Cryptococcus albidus, Saccharomyces cerevisiae*) that are or have been registered for the application as biocontrol products. These examples demonstrate the potential of yeasts for commercial biocontrol usage, but this review also highlights the scarcity of fundamental studies on yeast biocontrol mechanisms and of registered yeast-based biocontrol products. Yeast biocontrol mechanisms thus represent a largely unexplored field of research and plentiful opportunities for the development of commercial, yeast-based applications for plant protection exist.

**Electronic supplementary material:**

The online version of this article (10.1007/s11274-019-2728-4) contains supplementary material, which is available to authorized users.

## Introduction

Despite their relevance as model eukaryotes for biotechnological applications and in medical mycology, the potential use of antagonistic yeasts as biocontrol agents is still underexploited. Only a handful of yeast-based plant protection products has reached the market and even in fundamental research, antifungal yeasts have been neglected and poorly investigated with state-of-the-art technology and at the molecular level. Nevertheless, yeasts combine strong antifungal activities with advantageous properties for an application (e.g., strong antagonistic activity, culturability, formulatability, applicability, stress resistance) and are thereby promising for the development of biological plant protection agents. Furthermore, the close relatedness with model yeasts, particularly *Saccharomyces cerevisiae*, enables taking advantage of the molecular tools and plethora of data developed for these organisms for application-oriented and basic studies on biocontrol yeasts.

Biocontrol is mostly looked at and studied in a species/isolate-centric manner: different species/isolates are tested against the target plant pathogen and the most active organism is studied with respect to its potential for biocontrol applications. However, for a successful application and improvement of biocontrol organisms, we first have to understand the biocontrol mechanisms involved and then confirm their expression under field conditions (Droby and Chalutz 1994; Spadaro and Droby 2016; Wisniewski et al. 2007). Here, different yeast biocontrol mechanisms are highlighted and a comprehensive overview on published work on antagonistic mechanisms of biocontrol yeasts is provided in Supplementary Table 1.

## Advantageous yeast properties for potential biocontrol applications

Any organism to be used as the active ingredient in a biocontrol product must be effective against its target disease, but secondary properties such as biosafety and registration issues, production requirements and conditions, formulation options, and the required application equipment are just as or even more important. Although the lack of invasive, filamentous growth of most yeasts may seem a disadvantage, the yeast-like morphology is the reason for wieldy culturability in fermentors, advantageous formulation characteristics and ample application options. As for bacteria, the single-celled morphology of yeasts also favours adhesion and biofilm formation, which directly influences environmental persistence, competitiveness and thereby improved biocontrol activity (Fanning and Mitchell 2012; Pandin et al. 2017; Rossouw et al. 2018; Verstrepen and Klis 2006).

While many *S. cerevisiae* strains contain the high-copy 2 µm plasmid (i.e., 653 of 1011 sequenced *S. cerevisiae* strains) (Peter et al. 2018), most non-conventional yeasts lack plasmids (but can be engineered to maintain foreign, extra-chromosomal DNA by designing a plasmid vector containing intrinsic autonomously replicating and centromere sequences) (Cao et al. 2017). Yeasts thus share growth characteristics and biocontrol activities with bacteria without the risk of taking up or passing on plasmid-based antibiotic resistance, pathogenicity factors or toxin biosynthesis genes. In addition, horizontal gene transfer, albeit occurring more frequently in fungi than thought earlier, is significantly less frequent in yeasts, as compared to their prokaryotic counterparts, due to their more complex genome organisation (Fitzpatrick 2012; Moriguchi et al. 2013; Richards et al. 2011).

Yeasts have been used for food and beverage production for thousands of years, they are consumed directly as food supplements, and are widely employed in the food industry (Bekatorou et al. 2006; Querol and Fleet 2006). In many cases, these “food industry yeasts” belong to the same genus or even species as those intended for biocontrol (e.g., *S. cerevisiae*, *Candida sake*, *Metschnikowia pulcherrima*). This may be the reason why yeasts sensu lato are generally regarded as safe and therefore applying yeasts in crops and on food products elicits less concern than applications of bacteria or filamentous fungi (European Food Safety Authority 2005). Nonetheless, some yeasts, such as certain *Candida* or *Cryptococcus* species, are important fungal human pathogens (Butler et al. 2009; Miceli et al. 2011; Opulente et al. 2019). Properties such as dimorphism (e.g., the switch to an invasive growth form), growth at high temperatures (e.g., at or above 37 °C) and resistance to fungicides are of particular concern and should be studied and assessed in detail before considering new isolates for biocontrol applications (Gauthier 2015, 2017; Robert et al. 2015).

## Mechanisms underlying the biocontrol activity of yeasts

**Fig. 1:**
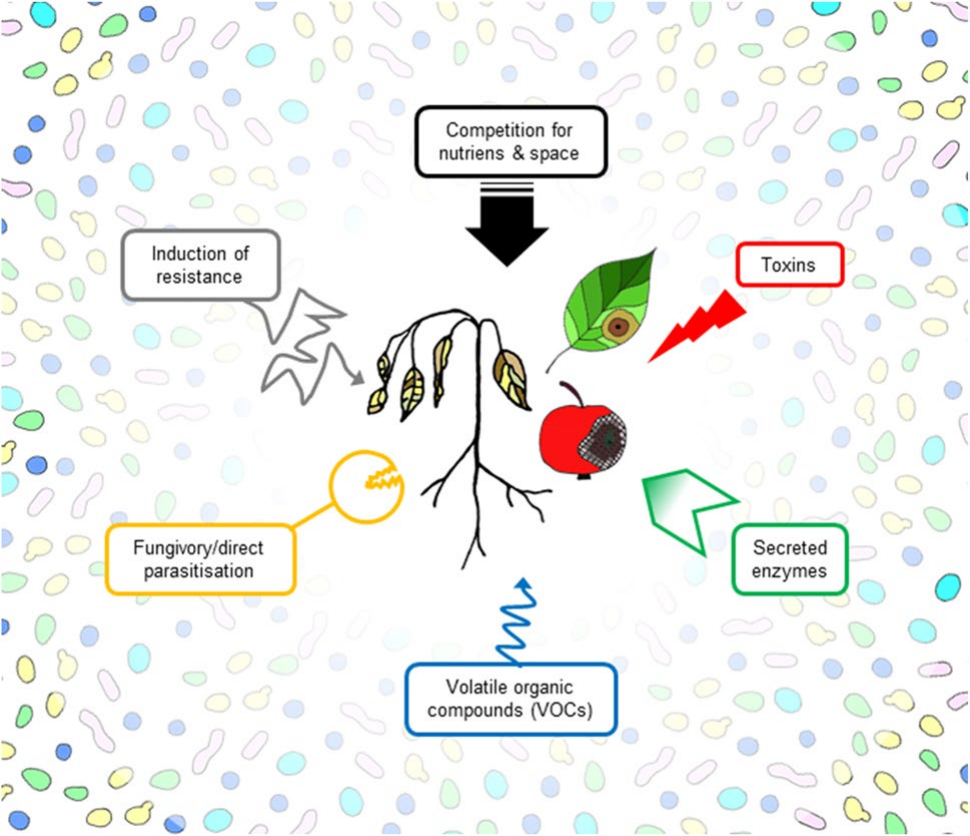
Multiple mechanisms are involved in preventing plant diseases and conferring biocontrol activity to yeasts. The mechanisms studied and highlighted here are competition for nutrients and space, secretion of toxins, enzymes and volatile organic compounds, direct parasitisation (fungivory) and indirect mechanisms (i.e., induction of resistance)

Understanding the mechanisms conferring biocontrol activity is the foundation for the informed and successful development and application of yeasts as plant protection agents (Droby and Chalutz 1994; Spadaro and Droby 2016; Wisniewski et al. 2007). For the biocontrol yeasts so far studied in detail, multiple mechanisms such as competition for nutrients and space, secretion of enzymes, toxin production, release of volatile organic compounds (VOCs), mycoparasitism and induction of resistance in plants are likely to be involved in the antagonistic function (Fig. 1) (Droby et al. 2009; Punja and Utkhede 2003; Wisniewski and Droby 2012). In most cases, the mechanisms outlined and discussed below have not been fully proven by molecular analyses (e.g., by gene deletion and complementation, heterologous expression), but rather proposed based on analogies with other biological systems. However, the increasing number of annotated yeast genomes and the availability of different transformation techniques should make it possible to decipher different mechanisms and to unambiguously confirm biocontrol mechanisms in future work.

### Competition for nutrients and space

All microorganisms compete with each other and their hosts for nutrients and space: this struggle is considered as the primary mode of action of biocontrol yeasts (Schaible and Kaufmann 2005; Spadaro and Droby 2016; Wisniewski et al. 2007). Competition is difficult to study mechanistically: it is likely more important in natural environments, where resources are limited and competitors plentiful. In community ecology, niche and nutrient competition have been intensely studied as determinants of species diversity. In nectar yeasts (e.g., *Metschnikowia reukaufii*), which are closely related to some biocontrol species, efficient resource depletion, due to the duplication of nitrogen transporter and metabolism genes, causes priority effects (i.e., order of species arrival determines community composition) and thus acts as a driver of competitiveness among different species (Dhami et al. 2016). With respect to competition for space, *in vitro* experiments performed on solid media seem to ascribe a minor role to a limitation in space. Although most yeasts grow well on agar plates, large differences in their antifungal activities were observed (Hilber-Bodmer et al. 2017). In addition, species-specific inhibition does not seem to occur, and a particular yeast is either strongly or weakly antagonistic against most fungi (Hilber-Bodmer et al. 2017). However, growing under field conditions activates diverse survival mechanisms and the competition for the physical niche might gain importance in such circumstances.

Most organisms and cells, from humans to bacteria, synthesise iron binding molecules to deprive competing organisms, pathogens or intracellular parasites of this essential element (Barber and Elde 2015; Johnson 2008). Also for biocontrol yeasts, iron is one of the most sought after nutrients and the competition for iron is recognised as an important mode of action (Spadaro and Droby 2016). In *Aureobasidium pullulans*, a siderophore identified as fusarinine C (fusigen) was identified and shown to exhibit antibacterial activity (Wang et al. 2009a, b). The peculiar red color of *M. pulcherrima* colonies is due to the formation of a cyclic dipeptide, pulcherriminic acid, that complexes iron (Gore-Lloyd et al. 2019). Pigmentless *M. pulcherrima* mutants were shown to exhibit reduced antifungal activity and iron deprivation of the fungal pathogen was suggested as one of several mechanisms by which this yeast antagonises plant pathogenic fungi (Gore-Lloyd et al. 2019; Sipiczki 2006). However, mutants lacking the ability to synthesise pulcherriminic acid still inhibited filamentous fungi strongly, suggesting that the antifungal activity was not only due to iron deprivation (Gore-Lloyd et al. 2019). The exact contribution of iron chelators to yeast biocontrol activity thus remains to be elucidated in detail.

Recently, it was shown that *Saccharomycopsis schoenii* lacks several components of the sulfur assimilation pathway and thus likely acquires methionine from its prey (Junker et al. 2019). Among yeasts, the inability to take up sulfur is specific to *Saccharomycopsis*, but some plant pathogenic fungi and *Trichoderma* species show a similar phenomenon, which may indicate that methionine is an important target for such organisms and highly competed over (Junker et al. 2019). Pioneering experiments aimed at evaluating the suitability of an easily transformable *Pichia (Ogataea) angusta* haploid strain to identify biocontrol-minus mutant clones: while the wild-type strain proved effective in reducing brown rot lesion caused by *Monilinia fructicola* on apple fruit, its derivate leucine-auxotrophic mutant L1 had no significant effect in controlling the pathogen. The addition of exogenous leucine fully restored the biocontrol capability of mutant L1, whereas a leucine stand-alone treatment showed no significant biocontrol effect (Fiori et al. 2008).

**Fig. 2:**
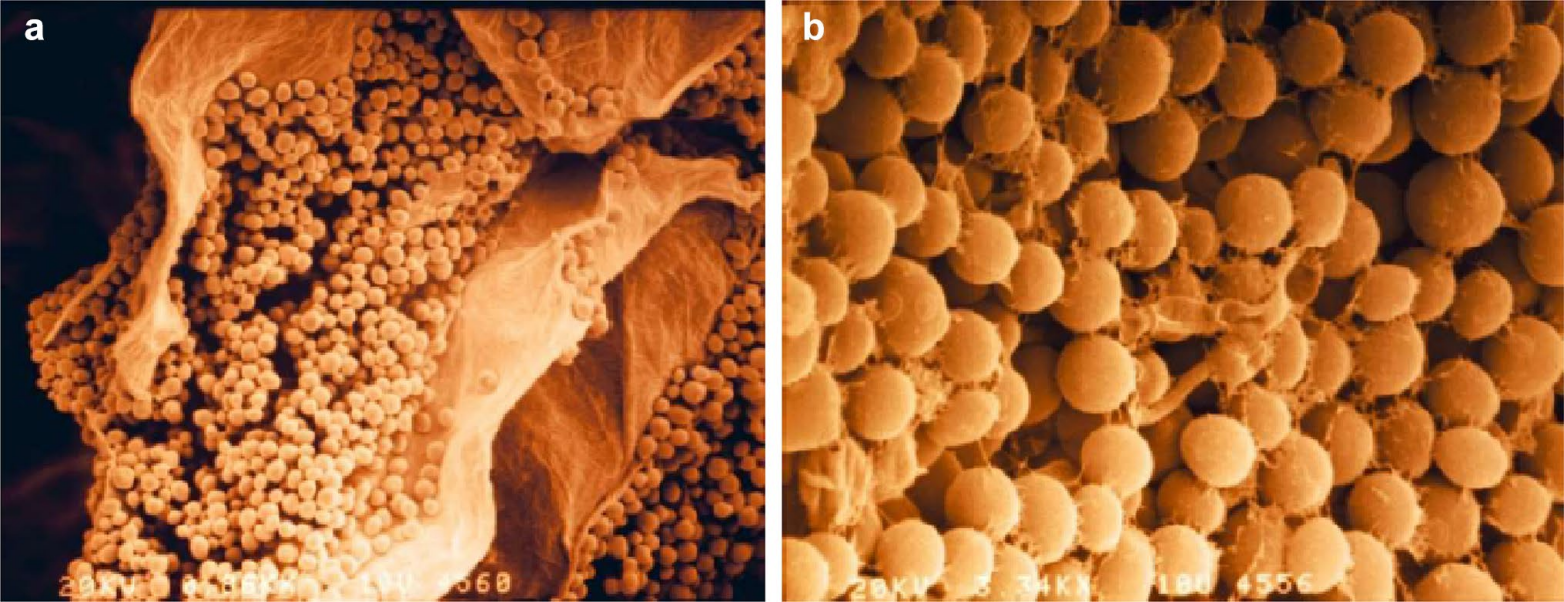
Colonisation **a** of the inner surface of an apple wound by the *Saccharomyces cerevisiae flor* strain M25. **b** *Penicilllium expansum* germ tubes grow onto the yeast cells, but contact with the apple tissue is prevented by a thick yeast cell layer. The presence of an extracellular matrix is likely to assure an effective protection of the apple tissue (Ortu et al. unpublished)

Biofilm formation may also be considered a specific and highly successful strategy to compete for space. Biofilms are microbial communities that live and grow on surfaces and can be comprised of a single species or represent multi-species consortia (Costa-Orlandi et al. 2017). Biofilms may exhibit vastly different properties as compared to free-floating cells and are considered a virulence factor for pathogenic microbes (Costa-Orlandi et al. 2017; Davey and O’Toole 2000; Desai et al. 2014). The development of a yeast biofilm starts with the adhesion of individual cells to a surface and usually involves cell wall modifications, secretion of an extracellular matrix, and often the formation of hyphae or pseudohyphae (Cavalheiro and Teixeira 2018; Costa-Orlandi et al. 2017). The process has been studied in detail and at the molecular level in medically relevant and model yeasts (Cabral et al. 2014; Cavalheiro and Teixeira 2018; d’Enfert and Janbon 2016; Lohse et al. 2018; Reynolds and Fink 2001). In biocontrol yeasts, biofilm formation, mainly in the phyllo- and carposphere (i.e., in wounds), is now considered an important mode of action and has been widely studied. However, the molecular underpinnings of the process and the composition of different biofilms (e.g., cell differentiation, multispecies biofilms) have only been studied in detail for *Pichia fermentans*. This species proved particularly intriguing in this respect, because biofilm formation in apple wounds protects against postharvest diseases, while on peaches *P. fermentans* switches from the yeast-like to the hyphal growth form and causes rapid decay of inoculated fruits in the absence of a plant pathogen (Fiori et al. 2012; Giobbe et al. 2007; Maserti et al. 2015; Sanna et al. 2012, 2013). Based on this “Jekyll & Hyde” pathogenic behaviour of *P. fermentans* on peach fruit, the capability to differentiate hyphae and pseudohyphae under particular growth conditions (e.g., depending on the nitrogen source) has been proposed as a potential biohazard factor for biocontrol yeasts (Giobbe et al. 2007). Besides *P.* *fermentans*, biofilm formation has also been implicated in the protective and biocontrol activities of *A. pullulans*, *Kloeckera apiculata*, *S. cerevisiae*, *Pichia kudriavzevii*, *W. anomalus*, and *M.* *pulcherrima* (Supplementary Table 1) (Chi et al. 2015; Klein and Kupper 2018; Ortu et al. 2005; Pu et al. 2014; Wachowska et al. 2016). In a *S. cerevisiae flor* strain, biofilm cells were far more efficient than planktonic cells in colonising the inner surface of apple wounds, thereby controlling the development of blue mould caused by *P. expansum* (Ortu et al. 2005; Scherm et al. 2003) (Fig. 2).

### Secreted enzymes

The secretion of enzymes degrading cellular components is a common feature in all kinds of host–pathogen interactions and has been intensively studied. Usually, such enzymes are upregulated in nutrient poor conditions and serve the provision of nutrients (e.g., carbon sources, amino acids) that are released from “prey” cells, which may lead to the killing of these cells (i.e., mycoparasitism/fungivory; see below). Secreted enzymes such as chitinases, glucanases, or proteases are thus regularly reported and highlighted in antagonistic yeasts and implicated in their biocontrol activity.

#### Chitinases

The secretion of chitinolytic enzymes is considered a desirable characteristic for biocontrol agents as it allows degrading fungal cell walls (Zajc et al. 2019). Chitin degrading activity has been measured in biocontrol yeasts of the genera *Aureobasidium, Candida, Debaryomyces, Metschnikowia*, *Meyerozyma*, *Pichia, Saccharomyces, Tilletiopsis*, and *Wickerhamomyces* and in *Saccharomycopsis*, chitinase expression was detected in the presence of prey cells (Supplementary Table 1) (Bar-Shimon et al. 2004; Junker et al. 2019; Lopes et al. 2015; Pretscher et al. 2018; Saravanakumar et al. 2009; Urquhart and Punja 2002; Zajc et al. 2019; Zhang et al. 2011). So far, the corresponding chitinase-encoding genes have not been cloned, deleted or overexpressed to unequivocally link these enzymes to biocontrol activity. However, chitinases from other sources than yeasts (i.e., filamentous fungi and bacteria) have demonstrated biocontrol activity against plant pathogenic fungi and chitinases are widely studied as potential biopesticides, targets for resistance breeding, or as transgenes in genetically modified plants (Dahiya et al. 2006; Herrera-Estrella and Chet 1999; Nagpure et al. 2014). Chitinases likely also affect biocontrol activity indirectly, because chito-oligosaccharides (CHOS; the results of chitin degradation) are potent inducers of plant immune responses (Kombrink et al. 2011; Langner and Gohre 2015; Liu et al. 2012, 2014).

#### Glucanases

Glucans are major cell wall components in fungi and exoglucanases are involved in cell wall modification, cell adhesion, and killer toxin resistance (Adams 2004; Jiang et al. 1995; Tsai et al. 2011; Xu et al. 2013). A 1,3-β-glucanase (*CoEXG1*) from *Candida oleophila* was the first gene cloned in this organism (Segal et al. 2002). Initial overexpression or deletion analyses of *CoEXG1* did not significantly affect spore germination of *Penicillium digitatum* (Yehuda et al. 2003), but later studies documented reduced inhibitory activity of the β-exoglucanase deletion mutant as compared to the wild type and overexpressing strain (*in vitro* and *in fructo*), thereby proving the involvement of glucanases in yeast biocontrol activity (Bar-Shimon et al. 2004). In *Wickerhamomyces anomalus* (*Pichia anomala*), the deletion of the two exo-β-glucanases (*PaEXG1* and *PaEXG2*) significantly reduced biocontrol activity on fruits against *Botrytis cinerea* (Friel et al. 2007), while the single deletion of *PaEXG2* did not reduce biocontrol performance (Grevesse et al. 2003). At the transcriptional level, differential upregulation of two *W. anomalus* (*P. anomala*) exoglucanase genes was shown during the interaction with plant pathogenic fungi on infected fruits or growth with fungal cell wall preparations (Parafati et al. 2017a). Exoglucanase activity has also been detected in numerous biocontrol yeasts (see Supplementary Table 1) (Chan and Tian 2005) and was linked to antagonistic activity, but without demonstrating a causal involvement. In *Rhodotorula glutinis* and *Cryptococcus laurentii*, β-1,3-glucanase activity did not correlate with the respective inhibitory activity against *B. cinerea* (Castoria et al. 1997). Six *S.* *cerevisiae* isolates exhibiting antifungal activity against *Colletotrichum acutatum* secreted exoglucanases, as did a *Pichia guilliermondi* biocontrol isolate (Lopes et al. 2015; Zhang et al. 2011).

#### Lipases

Lipolytic activity is frequently found when screening for extracellular enzymatic activity in yeast and yeast-like strains (Arroyo-Lopez et al. 2008; Buzzini and Martini 2002; Hernandez et al. 2007). This trait has been related to the consumption of previously accumulated lipids (in the so-called ‘oleaginous’ yeasts), and to cold tolerance in extremophilic yeasts (Białkowska and Turkiewicz 2014; Breuer and Harms 2006; Papanikolaou and Aggelis 2011; Szczęsna-Antczak et al. 2014). Besides this, lipase activity has been detected and shown to be involved in the pathogenicity of yeasts such as *Candida*, *Cryptococcus*, or *Malassezia* species (Mayer et al. 2013; Park et al. 2013; Sommer et al. 2016). Since a number of studies have highlighted the role of lipases in the biocontrol efficacy of bacteria and fungi against plant diseases and pests (Ali et al. 2009; Berto et al. 2001; Beys da Silva et al. 2010a, b; Keyhani 2018; Manuel et al. 2012; Sánchez-Pérez et al. 2014; Vial et al. 2007; Zha et al. 2014), the lipolytic activity of antagonistic yeasts may represent a promising target for innovative studies on biological control applications.

#### Proteases

Although proteases are important virulence factors in entomopathogenic fungi and filamentous mycoparasites, they have been scarcely studied in biocontrol yeasts (Supplementary Table 1). Since protease activity was only detected at later growth stages (after 6–8 days of growth in nutrient rich medium) in *C. oleophila* cultures, a minor function in biocontrol activity was hypothesised (Bar-Shimon et al. 2004). In contrast, the alkaline serine protease Alp5 from *A. pullulans* reduced spore germination and germ tube length of *Penicillium expansum*, *B. cinerea*, *M. fructicola* and *Alternaria alternata in vitro* and exhibited a concentration-dependent inhibitory effect on these pathogens on apple (Banani et al. 2014; Zhang et al. 2012). Protease activity has also been reported in the genera *Metschnikowia*, *Pichia*, and *Wickerhamomyces*, but not further studied or confirmed (Pretscher et al. 2018). Finally, *Saccharomycopsis* protease (and also glucanase) transcripts were significantly enriched during predation, but neither functionally investigated (Junker et al. 2019).

#### Toxin production

Yeasts are not known as prolific producers of secondary metabolites, which is one of the reasons why they often raise less biosafety concerns. Consequently, relatively few toxic molecules that may contribute to biocontrol activity have been described (Supplementary Table 1). Flocculosin is a low molecular weight cellobiose lipid produced by the biocontrol yeast *Pseudozyma flocculosa* (Mimee et al. 2005, 2009; Teichmann et al. 2011). *A. pullulans* (introduced in more detail below) produces diverse polymers (e.g., pullulan, aubasidan-like exopolysaccharide, poly(β-l-malic acid)), lipids, volatiles, enzymes, and secondary metabolites. Some of these metabolites (e.g., aureobasidins, liamocins, 2-propylacrylic acid, 2-methylenesuccinic acid) confer antagonistic activity against bacteria or fungi (Prasongsuk et al. 2018; Price et al. 2013, 2017; Takesako et al. 1991; Zain et al. 2009). Toxin production provided a competitive advantage to *A. pullulans* under dry, oligotrophic conditions, whereas it had no effect (as compared to yeasts not producing toxins) on antagonistic activity in more humid environments (McCormack et al. 1995). The most prominent toxins produced by many biocontrol yeast strains are proteinaceous killer toxins (Supplementary Table 1) (Bajaj et al. 2013; Banjara et al. 2016; Belda et al. 2017; Buzdar et al. 2011; Buzzini et al. 2004; Chen et al. 2000; Coelho et al. 2009; Comitini and Ciani 2011; Comitini et al. 2004; da Silva et al. 2008; De Ingeniis et al. 2009; Golubev et al. 2006; Guo et al. 2013; Guyard et al. 2002a, b; Hua et al. 2010; Kasahara 1994a, b; Klassen et al. 2004; Marquina et al. 2001; Ramirez et al. 2015; Rodriguez-Cousino et al. 2011; Santos and Marquina 2004a, b; Santos et al. 2002, 2009; Suzuki and Nikkuni 1994; Vepstaite-Monstavice et al. 2018; Wang et al. 2007, 2012a; Weiler and Schmitt 2003). These proteins were originally identified in *S. cerevisiae* and seem to mainly kill competing yeast species (Luksa et al. 2015; Schmitt and Breinig 2006). Yeast killer toxins have thus been mainly studied with respect to the control of spoilage yeasts in the beverage and food industry or for medical applications (Chessa et al. 2017; Chi et al. 2010; Lowes et al. 2000; Mannazzu et al. 2019; Schmitt and Breinig 2002). However, several of these toxins also inhibit or kill plant pathogenic fungi and were thus proposed for plant protection (Corbaci and Ucar 2018; Liu et al. 2015; Marquina et al. 2002; Perez et al. 2016). Nevertheless, further investigations to evaluate the specificity of yeast toxins and assess their effects on other beneficial microorganisms (e.g., in the phyllosphere, in soil microbiota and, in the case of edible commodities, the human gut) are required, particularly in the light of a possible registration.

#### Volatile organic compounds

Volatile organic compounds (VOCs) are small (usually < 300 Da) molecules with low water solubility and high vapour pressure. VOCs include a panoply of molecular classes, including hydrocarbons, alcohols, thioalcohols, aldehydes, ketones, thioesters, cyclohexanes, heterocyclic compounds, phenols and benzene derivatives (Morath et al. 2012). The chemical composition of each blend of volatiles (the so-called volatilome) may change depending on the producing yeast, the antagonised pathogen and the ecological niche where the cross-talking species are growing (Parafati et al. 2017b). Recent experimental evidence has revealed the key role of the yeast volatilome in yeast-pathogen interactions, including postharvest pathogens, and mycotoxin-producing fungi (Supplementary Table 1) (Bruce et al. 2003; Lemos Jr 2016; Parafati et al. 2015). Volatiles produced by *A. pullulans* proved efficient in reducing the growth and infection by *B. cinerea*, *C. acutatum, P.* *expansum, P. digitatum* and *P. italicum* both *in vitro* and *in planta* (Di Francesco et al. 2014). The biocontrol activity of different food yeasts such as *W. anomalus*, *M. pulcherrima*, *S. cerevisiae* and *A.* *pullulans* against *B. cinerea in vitro* and on table grape berries was largely attributed to the production of VOCs (Parafati et al. 2015). Similarly, VOCs released by *C. sake* reduced the incidence of apple rot caused by *P. expansum* and *B. cinerea* (Arrarte et al. 2017). The inhibitory activity of *Sporidiobolus pararoseus* on spore germination and mycelial growth of *B. cinerea* was mainly attributed to 2-ethyl-1-hexanol (Huang et al. 2012), whereas *Candida intermedia* produced 1,3,5,7-cyclooctatetraene, 3-methyl-1-butanol, 2-nonanone, and phenylethyl alcohol as the major components of its volatilome during the interaction with this pathogen (Huang et al. 2011). VOCs released by *P. anomala, Pichia kluyveri,* and *Hanseniaspora uvarum* inhibited *Aspergillus ochraceus* growth and ochratoxin A production during processing of coffee (Masoud et al. 2005), and 2-phenylethanol was identified as the key component of the *P. anomala* volatilome preventing spore germination, mycelial growth and toxin production by *Aspergillus flavus* (Hua et al. 2014). More than twenty different VOCs were identified in the volatilomes of selected biocontrol strains of *Cyberlindnera jadinii, Candida friedrichii, C. intermedia, and Lachancea thermotolerans*, but 2-phenylethanol was the most abundant and responsible for the inhibition of both mycelial growth and ochratoxin A production by *Aspergillus carbonarius* and *A. ochraceus* (Farbo et al. 2018; Fiori et al. 2014; Tilocca et al. 2019).

#### Mycoparasitism

Mycoparasitism (or fungivory, i.e., the consumption of one fungus by another) is rarely described and poorly studied in yeasts. *P. guilliermondii* was shown to strongly adhere to hyphae of the plant pathogen *B. cinerea* and to cause hyphal collapse, presumably due to the secretion of hydrolytic enzymes such as glucanases (see above) (Wisniewski et al. 1991). Similarly, the yeast-like Ustilaginomycete *Pseudozyma aphidis* parasitises the powdery mildew pathogen *Podosphaera xanthii* and *B. cinerea* (Calderon et al. 2019; Gafni et al. 2015). The genus *Saccharomycopsis*, comprising predacious yeasts directly feeding on their prey, was studied with respect to biocontrol of different *Penicillium* species as well as clinically relevant yeasts (Junker et al. 2017, 2018, 2019; Lachance and Pang 1997; Pimenta et al. 2008).

### Induction of resistance

Plants feature an innate immune system that recognises and responds to the presence of microorganisms (Chisholm et al. 2006; Jones and Dangl 2006). This plant immune response can induce resistance systemically and is the basis for the application of microorganisms as plant fertilisers and fortifiers (Gozzo and Faoro 2013; Pieterse et al. 2014). Biocontrol yeasts can elicit systemic resistance of plants against a broad range of pathogens (Supplementary Table 1) (Barda et al. 2015; Buxdorf et al. 2013a, b; Lee et al. 2017; Liu et al. 2016) and this activity is suggested to contribute to their biocontrol activity. For example, *S. cerevisiae*, *Rhodosporidium paludigenum*, *Candida saitoana*, *C. oleophila* and *Metschnikowia* species induce an innate immune response and eventually cause resistance against phyllosphere pathogens in fruits (De Miccolis Angelini et al. 2019; Droby et al. 2002; El Ghaouth et al. 2003; Hadwiger et al. 2015; Hershkovitz et al. 2012; Lu et al. 2013, 2014; Sun et al. 2018). In the case of *C. oleophila*, this induction has been attributed to the overproduction of reactive oxygen species in the plant (Macarisin et al. 2010), but yeast cell components (from dead cells) can also trigger systemic resistance (De Miccolis Angelini et al. 2019). Living cells are consequently not always required for such induction. In some cases, biocontrol yeasts such as *C. laurentii*, *Cryptococcus flavescens*, and *R. glutinis* have been used in combination with resistance inducers such as salicylic acid or rhamnolipids (Yan et al. 2014; Yu and Zheng 2006; Zhang et al. 2007c).

## Registered biocontrol yeast species

There is a huge discrepancy between the plethora of “biocontrol yeasts” described in scientific publications and the few yeast-based plant protection products that are registered and marketed as plant protection products. A range of factors (e.g., lack of mechanistic understanding, hurdles/costs of registration, lack of partners/consortia with required expertise, little commercial potential) are likely responsible for this apparent difficulty to develop yeast-based plant protection products. Here, we briefly highlight five yeast species (*C. oleophila, A. pullulans, M.* *fructicola, C. albidus,* and *S. cerevisiae*) that are currently or have been registered as plant protection agents.

### *Candida oleophila*

Species of the genus *Candida* are often isolated from environmental samples and many isolates strongly inhibit plant pathogens. Representatives are, for example, *C. diversa* (Li et al. 2016; Liu et al. 2017)*, C. ernobii* (Liu et al. 2010)*, C. guillermonidi* (McLaughlin et al. 1992; Papon et al. 2013; Saligkarias et al. 2002)*, C. oleophila* (Droby et al. 2002; Gamagae et al. 2003; Lahlali et al. 2004; Molinu et al. 2011; Wang et al. 2012b)*, C. saitoana* (Arras et al. 2006, 2010; El-Ghaouth et al. 1998, 2000a, b, c)*, C. sake* (Arrarte et al. 2017; Calvo-Garrido et al. 2013; Canamas et al. 2008; Carbo et al. 2018; McLaughlin et al. 1992; Morales et al. 2008; Nunes et al. 2002a, b; Torres et al. 2006; Usall et al. 2000; Yehuda et al. 2003), or *C. subhashii* (Hilber-Bodmer et al. 2017) that have all been envisioned as biocontrol agents against mold and postharvest diseases of pome, stone and citrus fruit. In particular for *C. sake*, a wealth of studies on production and formulation have been performed in order to render postharvest biocontrol more reliable and efficacious (Abadias et al. 2000, 2001a, b, c, 2003; Canamas et al. 2008; Carbo et al. 2018; Nunes et al. 2002a, b; Torres et al. 2003, 2006; Usall et al. 2000).

*C. oleophila* was the first yeast to be developed into a commercial plant protection agent and the fundamental research accompanying this initiative has established, for the first time, different mechanisms underlying the antifungal activity of yeasts in general. Although yeasts are generally believed to antagonise plant pathogenic fungi due to their competition for nutrients and space, the work on *C. oleophila* and other *Candida* species identified hydrolytic enzymes such as proteases, chitinases and glucanases, as well as volatile compounds, that have been implicated in antifungal activity (Bar-Shimon et al. 2004; Huang et al. 2011; Segal et al. 2002) (also see above and Supplementary Table 1). Furthermore, biofilm formation, high osmotolerance, induction of resistance in the plant/fruit, and direct parasitism of hyphae were shown to contribute to the biocontrol activity of *Candida* species (Droby and Chalutz 1994; Droby et al. 2002; El Ghaouth et al. 2003; Wisniewski et al. 1995, 2007). To overcome the inconsistent performance of the initial *Candida*-based biocontrol products (and of early biological plant protection products in general), combinations with fungicides, different buffers (e.g., calcium chloride, bicarbonate), chitosan, or lysozyme were studied (Droby et al. 1998, 2003a, b; El-Ghaouth and Wilson 2002; Scherm et al. 2003; Wilson and El-Ghaouth 2002). *C. oleophila* was also transformed, by electroporation and with the hygromycin B gene as a marker, to study its mode of antagonism at the molecular level (Yehuda et al. 2001).

The *C. oleophila* strains I-182 and O have been developed into the biocontrol products Aspire^®^ and Nexy^®^, respectively. The latter was the first biocontrol yeast to be registered against a postharvest disease (Wisniewski et al. 2007) and *C. oleophila* strain O has been approved as a plant protection agent in Europe in 2013 (European Commission Health & Consumers Directorate-General 2013; European Food Safety Authority (EFSA) 2015a).

### *Aureobasidium pullulans*

The saprophytic ascomycete *A. pullulans* is frequently isolated from leaf, flower or soil samples, occurs worldwide, and exhibits a polymorphic appearance. Biocontrol activity has been documented for several *A. pullulans* strains, but only DSM 14940 (CF 10) and DSM 14941 (CF 40) are registered, in mixture, as active ingredients of plant protection products against the fireblight disease caused by the bacterium *Erwinia amylovora* and postharvest diseases (European Food Safety Authority (EFSA) 2013). These two *A. pullulans* strains were selected based on their strong inhibition towards *E. amylovora* in co-culture experiments at high synthetic nectar concentration (25%) (Seibold et al. 2004). The two isolates DSM 14940 (CF 10) and DSM 14941 (CF 40) also exhibited stronger inhibitory activity, in detached flower assays, than other bacterial and yeast antagonists (Kunz 2004). The two strains were formulated as a wettable powder under the product name Blossom-Protect^®^ and tested under field conditions at different sites and over several years (Kunz 2004; Kunz and Haug 2006; Kunz et al. 2011; Seibold et al. 2004). The same two *A. pullulans* strains were also developed and registered to control postharvest diseases of apple as the product Boni-Protect^®^ (Weiss and Mögel 2006). Similar applications against storage and rot diseases of strawberries, plum and sour cherries are being studied (Holb and Kunz 2013; Weiss et al. 2014).

The two registered products containing *A. pullulans* as an active ingredient are interesting from different points of view. Contrary to the large majority of biocontrol products, Blossom- and Boni-Protect^®^ contain two different strains, albeit belonging to the same species. As for many other biocontrol yeasts, the *A. pullulans* mode of action involves competition for space and nutrients, but enzymes such as proteases, chitinases or secreted molecules (see above) may also be involved. Specific metabolites or enzymes and their contribution to the biocontrol activity of DSM 14940 (CF 10) and DSM 14941 (CF 40) have not been identified and the strains do not seem to have been characterised genetically. In contrast to most registered plant protection products, including biological products, the original Blossom-Protect^®^ has a rather limited range of application. The expansion to novel indications, beyond fireblight of pome fruit trees, is thus certainly also motivated by economic needs.

### *Metschnikowia fructicola*

The genus *Metschnikowia* comprises species of mainly phyllosphere and nectar yeasts that are globally distributed (Chappell and Fukami 2018; Lachance et al. 2001; Pozo et al. 2012; Slavikova et al. 2007; Vadkertiova et al. 2012). Among those, *M. fructicola* and *M. pulcherrima* are the most studied with respect to biocontrol, are able to inhibit a range of postharvest and plant rot diseases, and include the most potent antagonistic yeasts that have ever been identified (Akgun Karabulut et al. 2003; Hilber-Bodmer et al. 2017; Parafati et al. 2015; Piano et al. 1997; Saravanakumar et al. 2008; Spadaro et al. 2010a, b; Turkel et al. 2014). Complete genomes are available for several *Metschnikowia* species, including *M. fructicola* and *M. pulcherrima* (Gore-Lloyd et al. 2019; Piombo et al. 2018), and transformation protocols have been established and used to express green fluorescent protein and complement a naturally occurring mutant (Gore-Lloyd et al. 2019; Nigro et al. 1999).

The strong antifungal activity of *Metschnikowia* species is mediated by a range of mechanisms that involve competition for nutrients (e.g.; amino acids, iron), secretion of glucanases and chitinases, and the production of volatile organic compounds (Banani et al. 2015; Dhami et al. 2016; Gore-Lloyd et al. 2019; Hershkovitz et al. 2013; Saravanakumar et al. 2008; Sipiczki 2006; Zajc et al. 2019). The application of *Metschnikowia* cells to fruits (e.g., grapefruit) also induces an oxidative burst in the plant tissue that eventually results in the activation of plant defense responses (Hershkovitz et al. 2012; Macarisin et al. 2010).

Originally, *M. fructicola*, isolate NRRL Y-30752, was isolated and discovered in Israel and developed and registered as a biocontrol product for preventing postharvest diseases, particularly in sweet potato and carrot (Eshel et al. 2009; Kurtzman and Droby 2001; Wisniewski and Droby 2012). *M. fructicola* has also been patented as an antagonist of plant pathogenic microorganisms (Droby and El-Gerberia 2006). It seems that over time different companies showed interest in developing a biological fungicide based on *M. fructicola* NRRL Y-30752, but the isolate is now pursued by Koppert Biological Systems and has recently been approved as a plant protection agent against fungal diseases in stone fruits, strawberries and grapes by the European Food Safety Authority (EFSA) (European Food Safety Authority (EFSA) 2015c, 2017).

### *Cryptococcus albidus*

Basidiomycetes of the genus *Cryptococcus* are widespread in nature and frequently isolated from water sources, soil and decaying plant material. Studied for their potential to produce high lipid yields for biodiesel production, strains of *C. albidus, C. laurentii and C. flavus* have also been shown to protect peach, cherry, strawberry, tomato, citrus and pome fruits against postharvest decay (Elad et al. 1994; Tian et al. 2004; Zhang et al. 2007a, b). *C. albidus* was used as a biocontrol agent in the product Yieldplus^®^, which was registered in 1997 and marketed by Anchor Bio-Technologies in South Africa. This product was sold for over 15 years, but it has now been withdrawn from the market (Mbili 2012). Yieldplus^®^ was formulated for pome and citrus fruits against *B. cinerea* and *P. expansum* and later shown to be effective in the control of *Botrytis* during post-harvest cold storage of strawberries (Kowalska et al. 2012).

Regarding the mode of antagonism, most of the evidence points to competition for nutrients and space. Culture filtrates do not show any inhibitory activity against *B. cinerea* or *P. expansum*. However, both pathogens show reduced conidial germination and germ tube growth in liquid co-cultures (Fan and Tian 2001; Helbig 2002). The addition of glucose or NH_4_NO_3_ to the medium reduces biocontrol ability against *P. expansum*, but not against *B. cinerea* (Lutz et al. 2013). Beside nutrient competition, little conclusive evidence is available to determine a mode of antagonism. *C. albidus* exhibits glucanase, chitinase and protease activity in the corresponding substrate media. It also produces unidentified volatile compounds that inhibit fungal growth and can display killer activity against *C. glabrata* (Lutz et al. 2013). However, none of these mechanisms have been directly linked to the inhibitory activity of the target plant pathogens.

### *Saccharomyces cerevisiae*

*S. cerevisiae* is mainly known as a model organism for cell biology, its biotechnological usage, and most importantly the application in food and beverage production. Envisioning *S.* *cerevisiae* for biocontrol may be motivated by its perception as a safe organism that can be more easily registered, but also by its model organism status and the feasibility of molecular analyses. Overall, the model *S. cerevisiae* isolate BY4741 exhibited an intermediate antifungal activity against filamentous fungi and in comparison to a broad collection of wild yeast isolates (Hilber-Bodmer et al. 2017). This laboratory strain is thus ideally suited as a model host to express genes potentially involved in biocontrol activity and thereby improving or weakening its antifungal action.

A number of *S. cerevisiae* strains (e.g., DISAABA1182, RC008, RC009, RC012, and RC016) reduced the growth of plant pathogens such as *A. carbonarius*, *A. ochraceus*, *A. parasiticus* or *Fusarium graminearum* and also inhibited mycotoxin (e.g., aflatoxin, ochratoxin A, zearalenone, deoxynivalenol) production by these species (Armando et al. 2012a, 2013; Cubaiu et al. 2012). The mycotoxin-removing activity is due to adsorption to *S. cerevisiae* cell walls, stress responses to the toxin (e.g., changes in plasma membrane composition following patulin exposure), as well as direct transcriptional downregulation of polyketide synthesis (Armando et al. 2012b; Cubaiu et al. 2012; Oporto et al. 2019). Other biocontrol mechanisms employed by *S. cerevisiae* include the secretion of killer activity and hydrolytic enzymes as well as organic volatile compounds in yeasts that have been described and studied with respect to their antifungal activity against *C. acutatum* on citrus (Lopes et al. 2015) (also see Supplementary Table 1). Volatiles in general and specific compounds (e.g., alcohols and esters), were also identified in *S. cerevisiae* and implicated in the biocontrol activity against the citrus black spot disease caused by *Guignardia citricarpa* or postharvest decay in strawberries (Fialho et al. 2010; Oro et al. 2017). Seed, soil or foliar applications of a dried, active *S. cerevisiae* preparation also had a plant growth promoting effect and showed biocontrol activity against soilborne fungal pathogens such as *Fusarium*, *Sclerotium* or *Rhizoctonia* (Shalaby and El-Nady 2008). Finally, comprehensive transcriptome analyses have confirmed that the application of a cell wall preparation of the *S.* *cerevisiae* strain LAS117 (i.e., cerevisane^®^) induces the expression of genes involved in the plant response to fungal attack (De Miccolis Angelini et al. 2019). *S. cerevisiae* is therefore considered a promising biocontrol and probiotic organism for reducing growth of fungal pathogens and mycotoxins in fruit, vegetable and feedstuff (Cubaiu et al. 2012; Dogi et al. 2011; Pizzolitto et al. 2012; Prado et al. 2011). However, the only registered, active compound and commercial biocontrol application of Brewer’s yeast (*S. cerevisiae*) is the product Romeo^®^ with cerevisane^®^ as the active ingredient (European Food Safety Authority (EFSA) 2015b). This preparation is used as a preventive inducer of systemic resistance against powdery and downy mildew in grapes, fruits and vegetables and thus represents an application and plant protection product that differs from other such solutions insofar it is not based on active, living cells.

## Conclusions and outlook

The disparity between the number of yeast species exerting biocontrol activity against specific plant pathogens in laboratory assays and the number of yeast that are actually registered and successfully employed as plant protection products is likely caused by the lack of mechanistic understanding, the costs of registration, the lack of partners/consortia with required expertise, or a limited commercial potential. However, the general trend towards reduced pesticide use will certainly favour and create more incentives for the development of alternative plant protection solutions such as biocontrol yeasts. In the environment, these organisms interact intraspecifically, as well as with other microbes (including, but not exclusively, with plant pathogens) and host plants. These complex interactions and interdependencies eventually determine whether a disease sets in or an antagonistic yeast suppresses a pathogen and supports plant health. It is impossible to manage—for plant protection applications—such complex interactions without detailed knowledge of the interacting bionts. By studying and identifying modes of action in the laboratory, a reductionist approach is thus an important first step in the development and successful application of biocontrol in general.

Nowadays, the breakthrough achievements in the field of system biology, molecular biology and the related computational tools enable revealing the structural and functional peculiarities of any potential biocontrol agent. Exploiting these tools for the investigation and prediction of functional dynamics occurring between antagonists opens new avenues for the design of consortia of microbial antagonists that synergistically cooperate for the biocontrol of plant pathogens. Although strain mixtures for the biocontrol of plant pathogens are already available commercially (e.g., Blossom- and Boni-Protect®, see above) and described in the literature (Heydari and Pessarakli 2010; Lopes et al. 2012; Spadaro and Gullino 2005), employment of taxonomically divergent, but functionally complementary strains might represent a promising approach to follow in the near future in an attempt to design a standardised, multi-targeted, efficacious biocontrol strategy.

A deep mechanistic insight does not guarantee a successful product development and registration, but we argue that fundamental research on biocontrol mechanisms is a key aspect for successful biocontrol applications and at the same time still a frontier in biocontrol research. Hardly any antagonistic mechanism employed by biocontrol yeasts is understood and unequivocally proven by gene deletions and overexpression. This lack of fundamental understanding is also one of the reasons why, so far, little efforts were undertaken to improve, either by selection or molecular tools, biocontrol yeasts for plant protection. In general, molecular tools (e.g., gene deletion or overexpression, introduction of trans-genes, synthetic biology techniques) were rarely used in biocontrol yeasts, even though these technologies have the potential to empower studying and understanding these organisms at a whole new level (Marchand et al. 2007). Probably the first “biocontrol engineering example” among yeasts is a *S. cerevisiae* strain expressing the antifungal peptide cecropin A, which resulted in complete inhibition of *Colletotrichum coccodes* growth on tomatoes (Jones and Prusky 2002). Cecropin A was also expressed in *Pichia pastoris*, generating a strain controlling apple blue mold caused by *P. expansum* (Ren et al. 2012). A *P. pastoris* strain was also engineered for improved control of *P. expansum* by expressing the recombinant peach and pea defensins rDFN1 or rPsd1, respectively (Janisiewicz et al. 2008; Wisniewski et al. 2003). Besides this, only killer toxin activity has been extensively studied and transferred to new strains; mainly for biotechnological applications (Bajaj and Sharma 2010; Bussey et al. 1988; Schmitt and Schernikau 1997). Although the current legislation forbids the deliberate release of genetically manipulated microbials in the environment, model yeasts could also be engineered to shed light on the molecular mechanisms governing their antagonistic capability, their persistence on the host plant, or to better understand how to limit their capability to spread and interbreed (Callaway 2018; Goold et al. 2018; Klemsdal and Tronsmo 1999; Maselko et al. 2017).

Another frontier for biocontrol yeast research is the application in soil, for the protection against soilborne plant pathogens. Phyllosphere applications, particularly the use of yeasts against postharvest diseases, have been identified as the most promising target (Wisniewski et al. 2007). However, a broad range of yeast species occur predominantly in soil, exhibit strong antifungal activity, and could be envisioned as plant protection agents in this environment (Botha 2011; Hilber-Bodmer et al. 2017; Yurkov 2018). Developing yeasts as biocontrol agents against soilborne plant diseases is also attractive, because only few control options are available for the many severe soilborne plant diseases.

The development of a (yeast) biocontrol product depends on a chain of activities and disciplines, from science to industry and legislation, which have to come together, interact and build upon one another. Strengthening the exchange and interaction among these disciplines is thus essential to foster the commercialisation of biocontrol products (Usall et al. 2016). However, establishing such a virtuous cycle is difficult because of the different interests and qualities required and can be further hampered by economic constraints, commercial interests (and thereby a hesitation to share know-how or even material), or a lack of actors with complementary expertise. Considering the fact that many biocontrol solutions are local endeavours, have a limited potential to incur financial gains (in particular in comparison to medical applications), and are somehow idealistic in nature, commercial interests may actually rather harm than benefit the development of commercial biocontrol solutions. In particular, governmental research institutions, engaging in fundamental and applied research and being less driven by commercialisation, may take up a crucial spot for developing biocontrol solutions in the future.

## Electronic supplementary material

Below is the link to the electronic supplementary material.

Supplementary material 1 (PDF 475 kb)
