# Supplementary material for: Biocontrol yeasts: mechanisms and applications
Source: World J Microbiol Biotechnol. 2019 Oct 1;35(10):154. doi: 10.1007/s11274-019-2728-4 (PMC6773674; doi:10.1007/s11274-019-2728-4)
Supplement: Supplementary file 1 — Supplementary material 1 (PDF 475 kb) [file 11274_2019_2728_MOESM1_ESM.pdf]

Supplementary Table 1. Mechanisms underlying the antagonistic activity of yeasts. Different types of biocontrol mechanisms are divided into different specific mechanisms that were studied in distinct yeast species. Key studies and findings for all the specific mechanisms in different species are given without the claim for completeness. This overview is limited to studies that directly link specific properties/enzymes/metabolites to antibacterial or antifungal activity.

Type of mechanism	Specific mechanism	Species	References
**Competition for nutrients & space**	Rapid nitrogen/amino acid depletion	*Metschnikowia reukaufii*	Dhami et al. 2016
	General competition for nutrients	*Cryptococcus laurentii*	Castoria et al. 1997
		Rhodotorula glutinis	Castoria et al. 1997
	Iron deprivation	*Aureobasidium pullulans*	Wang et al. 2009a; Wang et al. 2009b
		Metschnikowia pulcherrima	Gore-Lloyd et al. 2019; Saravanakumar et al. 2008; Sipiczki 2006
	Sulfur metabolism	*Saccharomycopsis schoenii*	Junker et al. 2019
	Biofilm formation	*Aureobasidium pullulans*	Klein and Kupper 2018; Wachowska et al. 2016
		Kloeckera apiculata	Pu et al. 2014
		Metschnikowia pulcherrima	Parafati et al. 2015
		Pichia fermentans	Fiori et al. 2012; Giobbe et al. 2007; Maserti et al. 2015; Sanna et al. 2013; Sanna et al. 2012
		Pichia kudriavzevii	Chi et al. 2015
		Saccharomyces cerevisiae	Ortu et al. 2005; Scherm et al. 2003
		Wickerhamomyces anomalus	Parafati et al. 2015
**Secreted enzymes**	Chitinases	*Aureobasidium pullulans*	Zajc et al. 2019
		Candida oleophila	Bar-Shimon et al. 2004
		Cryptococcus albidus	Chan and Tian 2005

	Debaryomyces hansenii	Zajc et al. 2019
	Metschnikowia fructicola	Zajc et al. 2019
	Metschnikowia pulcherrima	Saravanakumar et al. 2009
	Meyerozyma guillermondii	Zajc et al. 2019
	Pichia guilliermondii	Zhang et al. 2011
	Pichia methanolica	Pretscher et al. 2018
	Pichia membranifaciens	Chan and Tian 2005
	Saccharomyces cerevisiae	Lopes et al. 2015
	Saccharomycopsis schoenii	Junker et al. 2019
	Tilletiopsis sp.	Urquhart and Punja 2002
	Wickerhamomyces anomalus	Pretscher et al. 2018
Glucanases	*Apiotrichum mycotoxinovorans*	Pretscher et al. 2018
	Candida oleophila	Bar-Shimon et al. 2004; Segal et al. 2002; Yehuda et al. 2003
	Cryptococcus albidus	Chan and Tian 2005
	Cryptococcus laurentii	Castoria et al. 1997
	Metschnikowia pulcherrima	Pretscher et al. 2018
	Pichia guilliermondii	Zhang et al. 2011
	Pichia membranifaciens	Chan and Tian 2005
	Pichia methanolica	Pretscher et al. 2018
	Rhodotorula glutinis	Castoria et al. 1997
	Saccharomyces cerevisiae	Lopes et al. 2015
	Saccharomycopsis schoenii	Junker et al. 2019
	Tilletiopsis sp.	Urquhart and Punja 2002

		Wickerhamomyces anomalus (Pichia anomala)	Friel et al. 2007; Grevesse et al. 2003; Parafati et al. 2017
	Lipases	Environmental isolates of ascomycete and basidiomycete yeasts	Arroyo-Lopez et al. 2008; Buzzini and Martini 2002; Hernandez et al. 2007
	Proteases	*Aureobasidium pullulans*	Banani et al. 2014; Zhang et al. 2012
		Candida oleophila	Bar-Shimon et al. 2004
		Metschnikowia pulcherrima	Pretscher et al. 2018
		Pichia methanolica	Pretscher et al. 2018
		Saccharomycopsis schoenii	Junker et al. 2019
		Wickerhamomyces anomalus	Pretscher et al. 2018
**Toxin production**	Killer toxin / Killer activity	*Candida guilliermondii*	Coelho et al. 2009
		Candida nodaensis	da Silva et al. 2008
		Debaryomyces hansenii	Banjara et al. 2016; Corbaci and Ucar 2018; Marquina et al. 2001; Santos et al. 2002
		Debaryomyces robertsiae	Klassen et al. 2004
		Kluyveromyces siamensis	Buzdar et al. 2011
		Kluyveromyces wickerhamii	Comitini and Ciani 2011
		Mrakia frigida	Hua et al. 2010
		Pichia acaciae	Klassen et al. 2004
		Pichia anomala	De Ingeniis et al. 2009; Wang et al. 2007
		Pichia farinosa	Suzuki and Nikkuni 1994
		Pichia kudriavzevii	Bajaj et al. 2013
		Pichia membranifaciens	Belda et al. 2017; Santos and Marquina 2004a; Santos and Marquina 2004b; Santos et al. 2009
		Pichia ohmeri	Coelho et al. 2009
		Pseudozyma tsukubaensis	Golubev et al. 2006

		Saccharomyces cerevisiae	Lopes et al. 2015; Rodriguez-Cousino et al. 2011
		Saccharomyces paradoxus	Vepstaite-Monstavice et al. 2018
		Schwanniomyces occidentalis	Chen et al. 2000
		Torulaspora delbrueckii	Ramirez et al. 2015; Villalba et al. 2016
		Tetrapisispora phaffii (Kluyveromyces phaffii)	Chessa et al. 2017; Comitini et al. 2004; Comitini et al. 2009
		Wickerhamomyces anomalus	Guo et al. 2013
		Williopsis saturnus	Buzzini et al. 2004; Guyard et al. 2002a; Guyard et al. 2002b; Wang et al. 2012
		Williopsis mrakii (Hansenula mrakii)	Kasahara et al. 1994a; Kasahara et al. 1994b; Lowes et al. 2000
		Zygosaccharomyces bailii	Weiler and Schmitt 2003
	Flocculosin	*Pseudozyma flocculosa*	Mimee et al. 2009; Mimee et al. 2005; Teichmann et al. 2011
	Aureobasidins	*Aureobasidium pullulans*	Takesako et al. 1991
	2-propylacrylic acid, 2-methylenesuccinic acid	*Aureobasidium pullulans*	Zain et al. 2009
	Liamocin	*Aureobasidium pullulans*	Price et al. 2017
	Unidentified toxins	*Tilletiopsis sp.*	Urquhart and Punja 2002
**Volatile organic compounds**	2-phenylethanol and others	*Aureobasidium pullulans*	Di Francesco et al. 2014
		Candida friedrichii	Farbo et al. 2018
		Candida intermedia	Farbo et al. 2018
		Cyberlindnera jadinii	Farbo et al. 2018
		Lachancea thermotolerans	Farbo et al. 2018
		Saccharomyces cerevisiae	Fialho et al. 2010

		Wickerhamomyces anomalus (Pichia anomala)	Hua et al. 2014
		Williopsis mrakii	Bruce et al. 2003
	2-phenylethyl acetate and others	*Hanseniaspora uvarum*	Masoud et al. 2005
		Pichia kluyveri	Masoud et al. 2005
	2-phenylethyl acetate and others	*Wickerhamomyces anomalus (Pichia anomala)*	Masoud et al. 2005
	2-ethyl-1-hexanol	*Sporidiobolus pararoseus*	Huang et al. 2012
	1,3,5,7-cyclooctatetraene, 3-methyl-1-butanol and others	*Candida intermedia*	Huang et al. 2011
	Unidentified volatiles	*Aureobasidium pullulans*	Parafati et al. 2015
		Candida sake	Arrarte et al. 2017
		Metschnikowia pulcherrima	Parafati et al. 2015
		Saccharomyces cerevisiae	Lopes et al. 2015; Oro et al. 2017; Parafati et al. 2015
		Starmerella bacillaris	Lemos et al. 2016
		Wicherhamomyces anomalus	Parafati et al. 2015
**Fungivory / direct parasitization**		*Sachharomycopsis schoenii*	Junker et al. 2018; Junker et al. 2019
		Pichia guillermondi	Wisniewski et al. 1991
		Pseudozyma aphidis	Calderon et al. 2019; Gafni et al. 2015
**Indirect mechanisms**	Induction of resistance	*Candida oleophila*	Droby et al. 2002
		Candida saitoana	El Ghaouth et al. 2003; Hadwiger et al. 2015
		Curibasidium pallidicorallinum	Hadwiger et al. 2015
		Kloeckera apiculata	Liu et al. 2016
		Metschnikowia fructicola	Hershkovitz et al. 2012

Metschnikowia pulcherrima	Hadwiger et al. 2015
Pseudozyma aphidis	Barda et al. 2015; Buxdorf et al. 2013a; Buxdorf et al. 2013b; Calderon et al. 2019; Gafni et al. 2015
Pseudozyma churashimaensis	Lee et al. 2017
Rhodosporidium paludigenum	Lu et al. 2014; Lu et al. 2013; Sun et al. 2018
Saccharomyces cerevisiae (including cell walls)	De Miccolis Angelini et al. 2019; Shalaby and El-Nady 2008

REFERENCES

Arrarte E, Garmendia G, Rossini C, Wisniewski M, Vero S (2017) Volatile organic compounds produced by Antarctic strains of *Candida sake* play a role in the control of postharvest pathogens of apples Biol Control 109:14-20 doi:10.1016/j.biocontrol.2017.03.002

Arroyo-Lopez FN, Querol A, Bautista-Gallego J, Garrido-Fernandez A (2008) Role of yeasts in table olive production Int J Food Microbiol 128:189-196 doi:10.1016/j.ijfoodmicro.2008.08.018

Bajaj BK, Raina S, Singh S (2013) Killer toxin from a novel killer yeast *Pichia kudriavzevii* RY55 with idiosyncratic antibacterial activity J Basic Microbiol 53:645-656 doi:10.1002/jobm.201200187

Banani H, Spadaro D, Zhang D, Matic S, Garibaldi A, Gullino ML (2014) Biocontrol activity of an alkaline serine protease from *Aureobasidium pullulans* expressed in *Pichia pastoris* against four postharvest pathogens on apple Int J Food Microbiol 182-183:1-8 doi:10.1016/j.ijfoodmicro.2014.05.001

Banjara N, Nickerson KW, Suhr MJ, Hallen-Adams HE (2016) Killer toxin from several food-derived *Debaryomyces hansenii* strains effective against pathogenic *Candida* yeasts Int J Food Microbiol 222:23-29 doi:10.1016/j.ijfoodmicro.2016.01.016

Bar-Shimon M et al. (2004) Characterization of extracellular lytic enzymes produced by the yeast biocontrol agent *Candida oleophila* Curr Genet 45:140-148 doi:10.1007/s00294-003-0471-7

Barda O, Shalev O, Alster S, Buxdorf K, Gafni A, Levy M (2015) *Pseudozyma aphidis* induces salicylic-acid-independent resistance to *Clavibacter michiganensis* in tomato plants Plant Dis 99:621-626 doi:10.1094/PDIS-04-14-0377-RE

Belda I, Ruiz J, Alonso A, Marquina D, Santos A (2017) The biology of *Pichia membranifaciens* killer toxins Toxins (Basel) 9 doi:10.3390/toxins9040112

Bruce A, Stewart D, Verrall S, Wheatley RE (2003) Effect of volatiles from bacteria and yeast on the growth and pigmentation of sapstain fungi Int Biodeterior Biodegradation 51:101-108 doi:10.1016/S0964-8305(02)00088-4

Buxdorf K, Rahat I, Gafni A, Levy M (2013a) The epiphytic fungus *Pseudozyma aphidis* induces jasmonic acid- and salicylic acid/nonexpressor of PR1-independent local and systemic resistance Plant Physiol 161:2014-2022 doi:10.1104/pp.112.212969

Buxdorf K, Rahat I, Levy M (2013b) *Pseudozyma aphidis* induces ethylene-independent resistance in plants Plant Signal Behav 8:e26273 doi:10.4161/psb.26273

Buzdar MA, Chi Z, Wang Q, Hua MX, Chi ZM (2011) Production, purification, and characterization of a novel killer toxin from *Kluyveromyces siamensis* against a pathogenic yeast in crab Appl Microbiol Biotechnol 91:1571-1579 doi:10.1007/s00253-011-3220-8

Buzzini P, Corazzi L, Turchetti B, Buratta M, Martini A (2004) Characterization of the *in vitro* antimycotic activity of a novel killer protein from *Williopsis saturnus* DBVPG 4561 against emerging pathogenic yeasts FEMS Microbiol Lett 238:359-365 doi:10.1016/j.femsle.2004.07.060

Buzzini P, Martini A (2002) Extracellular enzymatic activity profiles in yeast and yeast-like strains isolated from tropical environments J Appl Microbiol 93:1020-1025 doi:10.1046/j.1365-2672.2002.01783.x|
Calderon CE, Rotem N, Harris R, Vela-Corcia D, Levy M (2019) *Pseudozyma aphidis* activates reactive oxygen species production, programmed cell death and morphological alterations in the necrotrophic fungus *Botrytis cinerea* Mol Plant Pathol 20:562-574 doi:10.1111/mpp.12775
Castoria R, De Curtis F, Lima G, De Cicco V (1997) β-1,3-glucanase activity of two saprophytic yeasts and possible mode of action as biocontrol agents against postharvest diseases Postharvest Biol Technol 12:293-300 doi:10.1016/S0925-5214(97)00061-6
Chan Z, Tian S (2005) Interaction of antagonistic yeasts against postharvest pathogens of apple fruit and possible mode of action Postharvest Biol Technol 36:215-223 doi:10.1016/j.postharvbio.2005.01.001
Chen WB, Han YF, Jong SC, Chang SC (2000) Isolation, purification, and characterization of a killer protein from *Schwanniomyces occidentalis* Appl Environ Microbiol 66:5348-5352 doi:10.1128/aem.66.12.5348-5352.2000
Chessa R et al. (2017) Biotechnological exploitation of *Tetrapisispora phaffii* killer toxin: heterologous production in *Komagataella phaffii (Pichia pastoris*) Appl Microbiol Biotechnol 101:2931-2942 doi:10.1007/s00253-016-8050-2
Chi M et al. (2015) Increase in antioxidant enzyme activity, stress tolerance and biocontrol efficacy of *Pichia kudriavzevii* with the transition from a yeast-like to biofilm morphology Biol Cont 90:113-119 doi:10.1016/j.biocontrol.2015.06.006
Coelho AR, Tachi M, Pagnocca FC, Nobrega GM, Hoffmann FL, Harada K, Hirooka EY (2009) Purification of *Candida guilliermondii* and *Pichia ohmeri* killer toxin as an active agent against *Penicillium expansum* Food Addit Contam Part A Chem Anal Control Expo Risk Assess 26:73-81 doi:10.1080/02652030802227227
Comitini F, Ciani M (2011) *Kluyveromyces wickerhamii* killer toxin: purification and activity towards *Brettanomyces*/*Dekkera* yeasts in grape must FEMS Microbiol Lett 316:77-82 doi:10.1111/j.1574-6968.2010.02194.x
Comitini F, Di Pietro N, Zacchi L, Mannazzu I, Ciani M (2004) *Kluyveromyces phaffii* killer toxin active against wine spoilage yeasts: purification and characterization Microbiology 150:2535-2541 doi:10.1099/mic.0.27145-0
Comitini F, Mannazzu I, Ciani M (2009) *Tetrapisispora phaffii* killer toxin is a highly specific beta-glucanase that disrupts the integrity of the yeast cell wall Microb Cell Fact 8:55 doi:10.1186/1475-2859-8-55
Corbaci C, Ucar FB (2018) Purification, characterization and in vivo biocontrol efficiency of killer toxins from *Debaryomyces hansenii* strains Int J Biol Macromol 119:1077-1082 doi:10.1016/j.ijbiomac.2018.07.121
da Silva S, Calado S, Lucas C, Aguiar C (2008) Unusual properties of the halotolerant yeast *Candida nodaensis* killer toxin, CnKT Microbiol Res 163:243-251 doi:10.1016/j.micres.2007.04.002
De Ingeniis J, Raffaelli N, Ciani M, Mannazzu I (2009) *Pichia anomala* DBVPG 3003 secretes a ubiquitin-like protein that has antimicrobial activity Appl Environ Microbiol 75:1129-1134 doi:10.1128/AEM.01837-08
De Miccolis Angelini RM, Rotolo C, Gerin D, Abate D, Pollastro S, Faretra F (2019) Global transcriptome analysis and differentially expressed genes in grapevine after application of the yeast-derived defense inducer cerevisane Pest Manag Sci 75:2020-2033 doi:10.1002/ps.5317
Dhami MK, Hartwig T, Fukami T (2016) Genetic basis of priority effects: insights from nectar yeast Proc Biol Sci 283 doi:10.1098/rspb.2016.1455
Di Francesco A, Ugolini L, Lazzeri L, Mari M (2014) Production of volatile organic compounds by *Aureobasidium pullulans* as a potential mechanism of action against postharvest fruit pathogens Biol Control 81:8-14 doi:10.1016/j.biocontrol.2014.10.004
Droby S, Vinokur V, Weiss B, Cohen L, Daus A, Goldschmidt EE, Porat R (2002) Induction of resistance to *Penicillium digitatum* in grapefruit by the yeast biocontrol agent *Candida oleophila* Phytopathology 92:393-399 doi:10.1094/PHYTO.2002.92.4.393
El Ghaouth A, Wilson CL, Wisniewski M (2003) Control of postharvest decay of apple fruit with *Candida saitoana* and induction of defense responses Phytopathology 93:344-348 doi:10.1094/PHYTO.2003.93.3.344
Farbo MG et al. (2018) Effect of yeast volatile organic compounds on ochratoxin A-producing *Aspergillus carbonarius* and *A. ochraceus* Int J Food Microbiol 284:1-10 doi:10.1016/j.ijfoodmicro.2018.06.023
Fialho MB, Toffano L, Pedroso MP, Augusto F, Pascholati SF (2010) Volatile organic compounds produced by *Saccharomyces cerevisiae* inhibit the *in vitro* development of *Guignardia citricarpa*, the causal agent of citrus black spot World J Microb Biot 26:925-932 doi:10.1007/s11274-009-0255-4

Fiori S et al. (2012) Identification of differentially expressed genes associated with changes in the morphology of *Pichia fermentans* on apple and peach fruit FEMS Yeast Res 12:785-795 doi:10.1111/j.1567-1364.2012.00829.x
Friel D, Pessoa NM, Vandenbol M, Jijakli MH (2007) Separate and combined disruptions of two exo-β-1,3-glucanase genes decrease the efficiency of *Pichia anomala* (strain K) biocontrol against *Botrytis cinerea* on apple Mol Plant Microbe Interact 20:371-379 doi:10.1094/MPMI-20-4-0371
Gafni A, Calderon CE, Harris R, Buxdorf K, Dafa-Berger A, Zeilinger-Reichert E, Levy M (2015) Biological control of the cucurbit powdery mildew pathogen *Podosphaera xanthii* by means of the epiphytic fungus *Pseudozyma aphidis* and parasitism as a mode of action Front Plant Sci 6:132 doi:10.3389/fpls.2015.00132
Giobbe S, Marceddu S, Scherm B, Zara G, Mazzarello VL, Budroni M, Migheli Q (2007) The strange case of a biofilm-forming strain of *Pichia fermentans,* which controls *Monilinia* brown rot on apple but is pathogenic on peach fruit FEMS Yeast Res 7:1389-1398 doi:10.1111/j.1567-1364.2007.00301.x
Golubev WI, Pfeiffer I, Golubeva EW (2006) Mycocin production in *Pseudozyma tsukubaensis* Mycopathologia 162:313-316 doi:10.1007/s11046-006-0065-2
Gore-Lloyd D et al. (2019) Snf2 controls pulcherriminic acid biosynthesis and antifungal activity of the biocontrol yeast *Metschnikowia pulcherrima* Mol Microbiol 112:317-332 doi:10.1111/mmi.14272
Grevesse C, Lepoivre P, Jijakli MH (2003) Characterization of the exoglucanase-encoding gene *PaEXG2* and study of its role in the biocontrol activity of *Pichia anomala* strain K Phytopathology 93:1145-1152 doi:10.1094/PHYTO.2003.93.9.1145
Guo FJ, Ma Y, Xu HM, Wang XH, Chi ZM (2013) A novel killer toxin produced by the marine-derived yeast *Wickerhamomyces anomalus* YF07b Antonie Van Leeuwenhoek 103:737-746 doi:10.1007/s10482-012-9855-3
Guyard C, Dehecq E, Tissier JP, Polonelli L, Dei-Cas E, Cailliez JC, Menozzi FD (2002a) Involvement of [beta]-glucans in the wide-spectrum antimicrobial activity of *Williopsis saturnus var. mrakii* MUCL 41968 killer toxin Mol Med 8:686-694
Guyard C et al. (2002b) Characterization of a *Williopsis saturnus var. mrakii* high molecular weight secreted killer toxin with broad-spectrum antimicrobial activity J Antimicrob Chemother 49:961-971 doi:10.1093/jac/dkf040
Hadwiger LA, McDonel H, Glawe D (2015) Wild yeast strains as prospective candidates to induce resistance against potato late blight (*Phytophthora infestans*) Am J Potato Res 92:379-386 doi:10.1007/s12230-015-9443-y
Hernandez A, Martin A, Aranda E, Perez-Nevado F, Cordoba MG (2007) Identification and characterization of yeast isolated from the elaboration of seasoned green table olives Food Microbiol 24:346-351 doi:10.1016/j.fm.2006.07.022
Hershkovitz V et al. (2012) Global changes in gene expression of grapefruit peel tissue in response to the yeast biocontrol agent *Metschnikowia fructicola* Mol Plant Pathol 13:338-349 doi:10.1111/j.1364-3703.2011.00750.x
Hua MX, Chi Z, Liu GL, Buzdar MA, Chi ZM (2010) Production of a novel and cold-active killer toxin by *Mrakia frigida* 2E00797 isolated from sea sediment in Antarctica Extremophiles 14:515-521 doi:10.1007/s00792-010-0331-6
Hua SS, Beck JJ, Sarreal SB, Gee W (2014) The major volatile compound 2-phenylethanol from the biocontrol yeast, *Pichia anomala*, inhibits growth and expression of aflatoxin biosynthetic genes of *Aspergillus flavus* Mycotoxin Res 30:71-78 doi:10.1007/s12550-014-0189-z
Huang R, Che HJ, Zhang J, Yang L, Jiang DH, Li GQ (2012) Evaluation of *Sporidiobolus pararoseus* strain YCXT3 as biocontrol agent of *Botrytis cinerea* on post-harvest strawberry fruits Biol Control 62:53-63 doi:10.1016/j.biocontrol.2012.02.010
Huang R, Li GQ, Zhang J, Yang L, Che HJ, Jiang DH, Huang HC (2011) Control of postharvest *Botrytis* fruit rot of strawberry by volatile organic compounds of *Candida intermedia* Phytopathology 101:859-869 doi:10.1094/PHYTO-09-10-0255
Junker K, Bravo Ruiz G, Lorenz A, Walker L, Gow NAR, Wendland J (2018) The mycoparasitic yeast *Saccharomycopsis schoenii* predates and kills multi-drug resistant *Candida auris* Sci Rep 8:14959 doi:10.1038/s41598-018-33199-z
Junker K, Chailyan A, Hesselbart A, Forster J, Wendland J (2019) Multi-omics characterization of the necrotrophic mycoparasite *Saccharomycopsis schoenii* PLoS Pathog 15:e1007692 doi:10.1371/journal.ppat.1007692
Kasahara S et al. (1994a) Involvement of cell wall beta-glucan in the action of HM-1 killer toxin FEBS Lett 348:27-32
Kasahara S et al. (1994b) Cloning of the *Saccharomyces cerevisiae* gene whose overexpression overcomes the effects of HM-1 killer toxin, which inhibits beta-glucan synthesis J Bacteriol 176:1488-1499 doi:10.1128/jb.176.5.1488-1499.1994

Klassen R, Teichert S, Meinhardt F (2004) Novel yeast killer toxins provoke S-phase arrest and DNA damage checkpoint activation Mol Microbiol 53:263-273 doi:10.1111/j.1365-2958.2004.04119.x

Klein MN, Kupper KC (2018) Biofilm production by *Aureobasidium pullulans* improves biocontrol against sour rot in citrus Food Microbiol 69:1-10 doi:10.1016/j.fm.2017.07.008

Lee G, Lee SH, Kim KM, Ryu CM (2017) Foliar application of the leaf-colonizing yeast *Pseudozyma churashimaensis* elicits systemic defense of pepper against bacterial and viral pathogens Sci Rep 7:39432 doi:10.1038/srep39432

Lemos WJ, Jr. et al. (2016) Biocontrol ability and action mechanism of *Starmerella bacillaris* (synonym *Candida zemplinina*) isolated from wine musts against gray mold disease agent *Botrytis cinerea* on grape and their effects on alcoholic fermentation Front Microbiol 7:1249 doi:10.3389/fmicb.2016.01249

Liu P, Chen K, Li G, Yang X, Long CA (2016) Comparative transcriptional profiling of orange fruit in response to the biocontrol yeast *Kloeckera apiculata* and its active compounds BMC Genomics 17:17 doi:10.1186/s12864-015-2333-3

Lopes MR, Klein MN, Ferraz LP, da Silva AC, Kupper KC (2015) *Saccharomyces cerevisiae:* a novel and efficient biological control agent for *Colletotrichum acutatum* during pre-harvest Microbiol Res 175:93-99 doi:10.1016/j.micres.2015.04.003

Lowes KF, Shearman CA, Payne J, MacKenzie D, Archer DB, Merry RJ, Gasson MJ (2000) Prevention of yeast spoilage in feed and food by the yeast mycocin HMK Appl Environ Microbiol 66:1066-1076

Lu L, Xu S, Zeng L, Zheng X, Yu T (2014) *Rhodosporidium paludigenum* induced resistance in Ponkan mandarin against *Penicillium digitatum* requires ethylene-dependent signaling pathway Postharvest Biol Technol 97:93-101 doi:10.1016/j.postharvbio.2014.06.007

Lu L et al. (2013) Preharvest application of antagonistic yeast *Rhodosporidium paludigenum* induced resistance against postharvest diseases in mandarin orange Biol Control 67:130-136 doi:10.1016/j.biocontrol.2013.07.016

Marquina D, Barroso J, Santos A, Peinado JM (2001) Production and characteristics of *Debaryomyces hansenii* killer toxin Microbiol Res 156:387-391 doi:10.1078/0944-5013-00117

Maserti B, Podda A, Giorgetti L, Del Carratore R, Chevret D, Migheli Q (2015) Proteome changes during yeast-like and pseudohyphal growth in the biofilm-forming yeast *Pichia fermentans* Amino Acids 47:1091-1106 doi:10.1007/s00726-015-1933-1

Masoud W, Poll L, Jakobsen M (2005) Influence of volatile compounds produced by yeasts predominant during processing of *Coffea arabica* in East Africa on growth and ochratoxin A (OTA) production by *Aspergillus ochraceus* Yeast 22:1133-1142 doi:10.1002/yea.1304

Mimee B, Labbe C, Belanger RR (2009) Catabolism of flocculosin, an antimicrobial metabolite produced by *Pseudozyma flocculosa* Glycobiology 19:995-1001 doi:10.1093/glycob/cwp078

Mimee B, Labbe C, Pelletier R, Belanger RR (2005) Antifungal activity of flocculosin, a novel glycolipid isolated from *Pseudozyma flocculosa* Antimicrob Agents Chemother 49:1597-1599 doi:10.1128/AAC.49.4.1597-1599.2005

Oro L, Feliziani E, Ciani M, Romanazzi G, Comitini F (2017) Volatile organic compounds from *Wickerhamomyces anomalus*, *Metschnikowia pulcherrima* and *Saccharomyces cerevisiae* inhibit growth of decay causing fungi and control postharvest diseases of strawberries Int J Food Microbiol 265:18-22 doi:10.1016/j.ijfoodmicro.2017.10.027

Ortu G, Demontis MA, Budroni M, Goyard S, d'Enfert C, Migheli Q (2005) Study of biofilm formation in *Candida albicans* may help understanding the biocontrol capability of a *flor* strain of *Saccharomyces cerevisiae* against the phytopathogenic fungus *Penicillium expansum* J Plant Pathol 87:300

Parafati L, Cirvilleri G, Restuccia C, Wisniewski M (2017) Potential role of exoglucanase genes (*WaEXG1* and *WaEXG2*) in the biocontrol activity of *Wickerhamomyces anomalus* Microb Ecol 73:876-884 doi:10.1007/s00248-016-0887-5

Parafati L, Vitale A, Restuccia C, Cirvilleri G (2015) Biocontrol ability and action mechanism of food-isolated yeast strains against *Botrytis cinerea* causing post-harvest bunch rot of table grape Food Microbiol 47:85-92 doi:10.1016/j.fm.2014.11.013

Pretscher J et al. (2018) Yeasts from different habitats and their potential as biocontrol agents Fermentation 4:31 doi:10.3390/fermentation4020031

Price NP, Bischoff KM, Leathers TD, Cosse AA, Manitchotpisit P (2017) Polyols, not sugars, determine the structural diversity of anti-streptococcal liamocins produced by *Aureobasidium pullulans* strain NRRL 50380 J Antibiot (Tokyo) 70:136-141 doi:10.1038/ja.2016.92

Pu L, Jingfan F, Kai C, Chao-an L, Yunjiang C (2014) Phenylethanol promotes adhesion and biofilm formation of the antagonistic yeast *Kloeckera apiculata* for the control of blue mold on citrus FEMS Yeast Res 14:536-546 doi:10.1111/1567-1364.12139

Ramirez M, Velazquez R, Maqueda M, Lopez-Pineiro A, Ribas JC (2015) A new wine *Torulaspora delbrueckii* killer strain with broad antifungal activity and its toxin-encoding double-stranded RNA virus Front Microbiol 6:983 doi:10.3389/fmicb.2015.00983
Rodriguez-Cousino N, Maqueda M, Ambrona J, Zamora E, Esteban R, Ramirez M (2011) A new wine *Saccharomyces cerevisiae* killer toxin (Klus), encoded by a double-stranded rna virus, with broad antifungal activity is evolutionarily related to a chromosomal host gene Appl Environ Microbiol 77:1822-1832 doi:10.1128/AEM.02501-10
Sanna ML, Zara G, Zara S, Migheli Q, Budroni M, Mannazzu I (2013) A putative phospholipase C is involved in *Pichia fermentans* dimorphic transition Biochim Biophys Acta 1840:344-349 doi:10.1016/j.bbagen.2013.09.030
Sanna ML, Zara S, Zara G, Migheli Q, Budroni M, Mannazzu I (2012) *Pichia fermentans* dimorphic changes depend on the nitrogen source Fungal Biol 116:769-777 doi:10.1016/j.funbio.2012.04.008
Santos A, Marquina D (2004a) Ion channel activity by *Pichia membranifaciens* killer toxin Yeast 21:151-162 doi:10.1002/yea.1069
Santos A, Marquina D (2004b) Killer toxin of *Pichia membranifaciens* and its possible use as a biocontrol agent against grey mould disease of grapevine Microbiology 150:2527-2534 doi:10.1099/mic.0.27071-0
Santos A, Marquina D, Barroso J, Peinado JM (2002) (1-->6)-Beta-D-glucan as the cell wall binding site for *Debaryomyces hansenii* killer toxin Lett Appl Microbiol 34:95-99 doi:10.1046/j.1472-765x.2002.01053.x|
Santos A, San Mauro M, Bravo E, Marquina D (2009) PMKT2, a new killer toxin from *Pichia membranifaciens*, and its promising biotechnological properties for control of the spoilage yeast *Brettanomyces bruxellensis* Microbiology 155:624-634 doi:10.1099/mic.0.023663-0
Saravanakumar D, Clavorella A, Spadaro D, Garibaldi A, Gullino ML (2008) *Metschnikowia pulcherrima* strain MACH1 outcompetes *Botrytis cinerea*, *Alternaria alternata* and *Penicillium expansum* in apples through iron depletion Postharvest Biol Tec 49:121-128 doi:10.1016/j.postharvbio.2007.11.006
Saravanakumar D, Spadaro D, Garibaldi A, Gullino ML (2009) Detection of enzymatic activity and partial sequence of a chitinase gene in *Metschnikowia pulcherrima* strain MACH1 used as post-harvest biocontrol agent Eur J Plant Pathol 123:183-193 doi:10.1007/s10658-008-9355-5
Scherm B, Ortu G, Muzzu A, Budroni M, Arras G, Migheli Q (2003) Biocontrol activity of antagonistic yeasts against *Penicillium expansum* on apple J Plant Pathol 85:205-213
Segal E, Yehuda H, Droby S, Wisniewski M, Goldway M (2002) Cloning and analysis of CoEXG1, a secreted 1,3-β-glucanase of the yeast biocontrol agent *Candida oleophila* Yeast 19:1171-1182 doi:10.1002/yea.910
Shalaby ME-S, El-Nady MF (2008) Application of *Saccharomyces cerevisiae* as a biocontrol agent against *Fusarium* infection of sugar beet plants Acta Biologica Szegediensis 52:271-275
Sipiczki M (2006) *Metschnikowia* strains isolated from botrytized grapes antagonize fungal and bacterial growth by iron depletion Appl Environ Microb 72:6716-6724 doi:10.1128/Aem.01275-06
Sun C, Fu D, Lu H, Zhang J, Zheng X, Yu T (2018) Autoclaved yeast enhances the resistance against *Penicillium expansum* in postharvest pear fruit and its possible mechanisms of action Biol Control 119:51-58 doi:10.1016/j.biocontrol.2018.01.010
Suzuki C, Nikkuni S (1994) The primary and subunit structure of a novel type killer toxin produced by a halotolerant yeast, *Pichia farinosa* J Biol Chem 269:3041-3046
Takesako K et al. (1991) Aureobasidins, new antifungal antibiotics. Taxonomy, fermentation, isolation, and properties J Antibiot (Tokyo) 44:919-924 doi:10.7164/antibiotics.44.919
Teichmann B, Labbe C, Lefebvre F, Bolker M, Linne U, Belanger RR (2011) Identification of a biosynthesis gene cluster for flocculosin a cellobiose lipid produced by the biocontrol agent *Pseudozyma flocculosa* Mol Microbiol 79:1483-1495 doi:10.1111/j.1365-2958.2010.07533.x
Urquhart EJ, Punja ZK (2002) Hydrolytic enzymes and antifungal compounds produced by *Tilletiopsis* species, phyllosphere yeasts that are antagonists of powdery mildew fungi Can J Microbiol 48:219-229 doi:10.1139/w02-008
Vepstaite-Monstavice I et al. (2018) *Saccharomyces paradoxus* K66 killer system evidences expanded assortment of helper and satellite viruses Viruses 10 doi:10.3390/v10100564
Villalba ML, Susana Saez J, Del Monaco S, Lopes CA, Sangorrin MP (2016) TdKT, a new killer toxin produced by *Torulaspora delbrueckii* effective against wine spoilage yeasts Int J Food Microbiol 217:94-100 doi:10.1016/j.ijfoodmicro.2015.10.006

Wachowska U, Głowacka K, Mikołajczyk W, Kucharska K (2016) Biofilm of *Aureobasidium pullulans* var. *pullulans* on winter wheat kernels and its effect on other microorganisms Microbiology 85:523-530 doi:10.1134/S0026261716050192
Wang W, Chi Z, Liu G, Buzdar MA, Chi Z, Gu Q (2009a) Chemical and biological characterization of siderophore produced by the marine-derived *Aureobasidium pullulans* HN6.2 and its antibacterial activity Biometals 22:965-972 doi:10.1007/s10534-009-9248-x
Wang WL, Chi ZM, Chi Z, Li J, Wang XH (2009b) Siderophore production by the marine-derived *Aureobasidium pullulans* and its antimicrobial activity Bioresour Technol 100:2639-2641 doi:10.1016/j.biortech.2008.12.010
Wang X, Chi Z, Yue L, Li J (2007) Purification and characterization of killer toxin from a marine yeast *Pichia anomala* YF07b against the pathogenic yeast in crab Curr Microbiol 55:396-3401 doi:10.1007/s00284-007-9010-y
Wang XX, Chi Z, Peng Y, Wang XH, Ru SG, Chi ZM (2012) Purification, characterization and gene cloning of the killer toxin produced by the marine-derived yeast *Williopsis saturnus* WC91-2 Microbiol Res 167:558-563 doi:10.1016/j.micres.2011.12.001
Weiler F, Schmitt MJ (2003) Zygocin, a secreted antifungal toxin of the yeast *Zygosaccharomyces bailii*, and its effect on sensitive fungal cells FEMS Yeast Res 3:69-76 doi:10.1016/s1567-1356(02)00126-5
Wisniewski M, Biles C, Droby S, Mclaughlin R, Wilson C, Chalutz E (1991) Mode of action of the postharvest biocontrol yeast, *Pichia guilliermondii* .1. Characterization of attachment to *Botrytis cinerea* Physiol Mol Plant P 39:245-258 doi:10.1016/0885-5765(91)90033-E
Yehuda H, Droby S, Bar-Shimon M, Wisniewski M, Goldway M (2003) The effect of under- and overexpressed CoEXG1-encoded exoglucanase secreted by *Candida oleophila* on the biocontrol of *Penicillium digitatum* Yeast 20:771-780 doi:10.1002/yea.1006
Zain M, Awaad A, A. Razzak A, Maitland D, El-Sayed N, Sakhawy M (2009) Secondary metabolites of *Aureobasidium pullulans* isolated from egyptian soil and their biological activity Journal of Applied Sciences Research 5:1582-1591
Zajc J, Gostincar C, Cernosa A, Gunde-Cimerman N (2019) Stress-tolerant yeasts: opportunistic pathogenicity versus biocontrol potential Genes (Basel) 10:42 doi:10.3390/genes10010042
Zhang D, Spadaro D, Garibaldi A, Gullino ML (2011) Potential biocontrol activity of a strain of *Pichia guilliermondii* against grey mold of apples and its possible modes of action Biol Control 57:193-201 doi:10.1016/j.biocontrol.2011.02.011
Zhang D, Spadaro D, Valente S, Garibaldi A, Gullino ML (2012) Cloning, characterization, expression and antifungal activity of an alkaline serine protease of *Aureobasidium pullulans* PL5 involved in the biological control of postharvest pathogens Int J Food Microbiol 153:453-464 doi:10.1016/j.ijfoodmicro.2011.12.016
